# Tumor volume changes after stereotactic, hypofractionated and conventional radiotherapy in paragangliomas of head and neck

**DOI:** 10.1002/cam4.70232

**Published:** 2024-09-13

**Authors:** Paweł J. Polanowski, Agnieszka R. Kotecka‐Blicharz, Andrzej Tukiendorf, Natalia J. Amrogowicz, Aleksandra M. Nasiek, Agnieszka Pietruszka, Katarzyna M. Polanowska, Krzysztof A. Składowski

**Affiliations:** ^1^ First Radiation and Clinical Oncology Department Maria Sklodowska‐Curie National Research Institute of Oncology Gliwice Poland; ^2^ Department of Nuclear Medicine and Endocrine Oncology Maria Sklodowska‐Curie National Research Institute of Oncology Gliwice Poland; ^3^ Institute of Health Sciences, Opole University Opole Poland; ^4^ Department of Clinical Oncology Maria Sklodowska‐Curie National Research Institute of Oncology Cracow Poland; ^5^ Ophthalmology Department St. Barbara Provincial Hospital No 5 Sosnowiec Poland

**Keywords:** conventional radiotherapy, head and neck, hypofractionated radiotherapy, paragangliomas, stereotactic radiotherapy

## Abstract

**Background:**

The aim of this study is comparison the effectiveness of stereotactic, hypofractionated and conventional radiotherapy assessed by the tumor volume changes of paraganglioma located in the head and neck region concerning fractional and total doses.

**Methods:**

We analyzed 76 patients after radiotherapy due to paraganglioma who were assigned to 3 groups considering fractional (≤2 Gy, 3–5.5 Gy, ≥6 Gy) and total (≤20 Gy, 21–40 Gy, >40 Gy) doses. The volumes of irradiated tumors were measured and compared based on diagnostic images performed before and after the treatment.

**Results:**

The mean tumor volume after the treatment with the lowest fractional dose (≤2 Gy) was decreased by 14.4 cm^3^. In patients treated with higher fractional doses (>2 Gy), the mean tumor volumes decreased by less than 1 cm^3^ for hypofractionated and stereotactic radiotherapy. 15.9 cm^3^ reduction of the mean tumor volume after the treatment with the highest RT total dose (>40 Gy) was stated. In patients treated with total doses ≤20 Gy and 21–40 Gy, the mean tumor volume was stable and reduced by 1.15 cm^3^, respectively. The analysis demonstrates a statistically significant (*p* < 0.05) treatment advantage in patients after the lowest fractional and highest total doses.

**Conclusion:**

The reduction of the tumor's volume was reported after conventional and unconventional radiotherapy. The most significant depletion of the paraganglioma volume was noted after a factional dose ≤2 Gy and a total dose >40 Gy.

## 1 INTRODUCTION

Paragangliomas (PGs) are rare, typically benign tumors arising from chromaffin neuroendocrine cells.^1^ It is estimated that about 3% of all paragangliomas are located in the head and neck (H&N) region, accounting for 0.6% of all tumors in this anatomical area.^2^, ^3^, ^4^, ^5^ Commonly paragangliomas are found at the bifurcation of the common carotid artery, in the middle ear, nearby the jugular foramen and along the vagal nerve. The larynx, nasopharynx or orbit are casuistic locations.^6^, ^7^ Middle‐aged females are more predisposed to the occurrence of H&N PGs.^8^, ^9^ These tumors are slow‐growing (1–5 mm per year),^10^, ^11^ encapsulated and hypervascular which is the reason for the avoidance of the biopsy due to the risk of hemorrhage.^12^ The malignant behavior of paraganglioma is defined as metastasis to non‐neuroendocrine tissue. Most metastases are located in regional lymph nodes or distant destinations like lungs, bones or liver.^5^, ^13^ Malignant paragangliomas may account for from 4% (jugulotympanic and carotid PGs) to 16% (vagal PGs) of all PGs.^14^, ^15^ Predominantly PGs are single lesions, even though 15% might be presented as multifocal.^16^, ^17^ The higher proportion of multifocal is related to hereditary disease and reaches up to 40% in the group of familial paragangliomas,^18^ which represent 10%–50% of all PGs.^19^ The most common mutations responsible for the development of H&N PGs involve gene coding subunits of succinate dehydrogenase.^20^, ^21^ In rare cases, mutations in genes such as VHL, NF1, RET, TMEM127, and MAX may predispose to H&N PGs development.^22^ Mass effect, hoarseness, tinnitus, hearing loss, or cranial nerve palsies are popular symptoms of PGs in H&N.^23^, ^24^ Some patients also report headaches, hypertension, cardiac rhythm disorder or excess sweating. The revealing of these symptoms may indicate secretory paragangliomas posing 5% of H&N PGs.^25^, ^26^ The crucial in the diagnostic process of PGs is magnetic resonance imaging (MRI) revealing strongly enhanced tumor with salt and pepper appearance, and angiography showing vessels building tumor mass. Positron emission tomography with 68 Ga‐DOTA‐conjugated somatostatin receptor‐targeting peptide facilitates distinguishing metastatic lesions from multifocal cases.^27^, ^28^, ^29^, ^30^ A decision concerning therapeutic method should be made on the H&N unit due to an array of possibilities after taking account of the patient's age, comorbidities and localization of the tumor. Elder patients with serious concomitant diseases who do not report symptoms of paraganglioma may need only active observation using imaging methods thereby avoiding operation.^31^ Surgery, except for hard‐to‐reach PGs located in a skull base, is the most radical method of treatment but is burdened with the high risk of hemorrhagic or neuropathic complications. To decrease the risk of bleeding, a preoperative embolization is recommended nonetheless associated with the risk of a stroke.^32^ Another, noninvasive treatment option is radiotherapy (RTH). Tumors <3 cm are suitable for stereotactic radiotherapy or radiosurgery with application 21–25 Gy in 3–5 fractions and 12–30 Gy in one fraction, respectively. Qualification to conventional radiotherapy does not require specific tumor size. The most common prescribed dose is 40–50 Gy in 20–25 fractions. Stagnation or partial regression are usually observed after radiotherapy but literature data does not indicate explicitly the most effective scheme.^33^, ^34^, ^35^ In metastatic paraganglioma systemic treatment encompasses chemotherapy, radionuclide therapy or targeted therapy. Due to the expression of somatostatin receptor type 2 and norepinephrine transporter in chromaffin cells radioisotope therapy with radiolabeled somatostatin or 131I‐MIBG may be an efficacious option in disseminated disease. At this point, consideration of radiotherapy may also represent a reasonable method in selected oligo‐metastatic cases with mass effect symptoms.^36^, ^37^ The main goal of this work is the assessment of the efficacy of radiotherapy measured by tumor volume changes taking into account fractional and total dose.

## 2 MATERIALS AND METHODS

This paper is a retrospective and single‐institution study conducted at Maria Sklodowska‐Curie National Research Institute of Oncology, Gliwice Branch in Poland. We identified 76 patients treated between 2008 and 2020. The characteristic of patients is presented in Table 1. Nine patients had bilateral paragangliomas, one had ipsilateral two paragangliomas, and one had paraganglioma with metastasis to the central nervous system. A single paraganglioma was found in other patients. Three patients were irradiated two times due to progression after the first radiotherapy. Two of them were treated with 3 × 6 Gy and this scheme was repeated after progression, gaining stabilization. One patient had progression after 4 × 5 Gy and received 10 × 2 Gy also getting stabilization. Aggregatively, 85 paragangliomas were analyzed in the whole group of patients. Twenty‐seven paragangliomas were operated on before radiotherapy—7 were incompletely resected and hence needed adjuvant RTH, other 20 were completely resected but due to recurrence, RTH was required. No patient was operated on after radiotherapy. Fourteen paragangliomas were submitted embolization (13 embolizations with insufficient effect before radiotherapy). Only one patient with laryngeal paraganglioma needed embolization due to the escalation of hemorrhage after the second fraction of 6 Gy. Stagnation was confirmed after this combined treatment. During radiotherapy, every patient was immobilized with thermoplastic masks. Computed tomography scans (3–5 mm slice thickness) with or without intravenous contrast (physician decisions) were performed in the supine position in planning the radiotherapy process. MRI scan with gadolinium intravenous contrast as supportive imaging was conducted on 69 patients. The physician defined gross tumor volume (GTV) and created clinical target volume (CTV) by adding a 1–3 mm margin to GTV. In the next step planning target volume was generated adding a 2–3 mm margin to CTV. In the case of stereotactic radiotherapy creating of CTV was omitted. The main goal of this work was to assess the change in the volume of paraganglioma after using different methods of radiotherapy. The initial volume of the tumor was founded on diagnostic imaging performed before treatment. The last imaging which was registered at the time of follow‐up was used to figure out the final volume of the tumor. Volumes were calculated based on three dimensions of each paraganglioma. To standardize different schemes of treatment we assigned patients to three groups in terms of fractional (≤2 Gy, 3–5.5 Gy, ≥6 Gy) and total (≤20 Gy, 21–40 Gy, >40 Gy) doses. Follow‐up was 157–3665 days (mean 1116). Four patients did not report for follow‐up.

**TABLE 1:** Patient and treatment characteristics.

Age	16–80 years (mean 52)
Sex	56 female
	20 male
Number of analyzed paragangliomas	85
Localization	Number of paragangliomas
Middle ear (jugulotympanic)	46 (+3 reirradiated)
Carotid body	32
Vagal	3
Laryngeal	1
Symptoms	Number of patients
Palpable tumor	17
Tinnitus	21
Hypoacusis	28
Dizziness	19
Palsy of *n*. VII, IX, X, XI, or XII	12
Tumor hemorrhage	2
Without symptoms	9
Genetic mutation	
VHL	1
SDHA	1
SDHB	7
SDHC	3
SDHD	6
Technique of radiotherapy	
IMRT	5
VMAT	18
Cyber knife	56
Static 3D‐conformal	6
Toxicity	
Acute	
Skin	6 (G1)
Mucosa	9 (G1)
Late	
Skin	8 (G1)
Mucosa	0

### 2.1 Statistical analysis

To determine the effect of two nominal predictor variables (the period before and after RTH and the RTH method) on a continuous outcome variable (tumor volume), a two‐way analysis of variance (ANOVA) test as a statistical test was used. In particular, conventional two‐way ANOVA analyzes the effect of the independent variables on the expected outcome (described as main effects) along with their relationship to the outcome itself (known as interaction effects). Factor interaction refers to the case where each factor not only exerts an effect on the response variable, but also may interact with the other factor to exert additional joint effects on the response variable (it tests the effect of the combination of two factors at the same time). Additionally, to specify differences of means between factor combinations, pairwise contrasts between the groups were conducted.

## 3 RESULTS

The descriptive statistics of tumor volume (mean, standard deviation = SD, median, and range) by RTH method, fractional dose, total dose, and observation period are given in Table 2.

**TABLE 2:** Tumor volume (cm^3^) by RTH method, fractional dose, total dose, and observation period.

	Number of paragangliomas		Period	Mean	SD	Median	Minimum	Maximum
RTH method	21	Conventional	Before	30.5	59.6	14.2	0.11	283
		Conventional	After	15	13.5	12.1	0.11	44.5
	64	Unconventional	Before	12.2	12	9.24	0.04	56.1
		Unconventional	After	11.3	12.1	8.01	0.04	56.1
Fractional dose	21	≤2Gy	Before	31.4	57.1	17.4	0.11	283
		≤2Gy	After	17	15.9	12.4	0.11	56.1
	13	3–5.5Gy	Before	18	14.1	13.4	2.35	42.5
		3–5.5Gy	After	17.6	16.3	10.5	1.67	56.1
	51	>6Gy	Before	9.51	9.08	7.24	0.04	40.6
		>6Gy	After	8.81	8.15	7.84	0.04	34.2
Total dose	29	≤20Gy	Before	14.7	12.4	12.6	0.89	56.1
		≤20Gy	After	14.8	14	10.8	0.15	56.1
	37	21–40Gy	Before	9.4	11.2	5.52	0.04	42.5
		21–40Gy	After	8.25	9.54	5.03	0.04	37.9
	19	>40Gy	Before	31.7	59.3	17.4	0.11	283
		>40Gy	After	15.8	13.4	12.4	0.11	44.5

**FIGURE 1:**
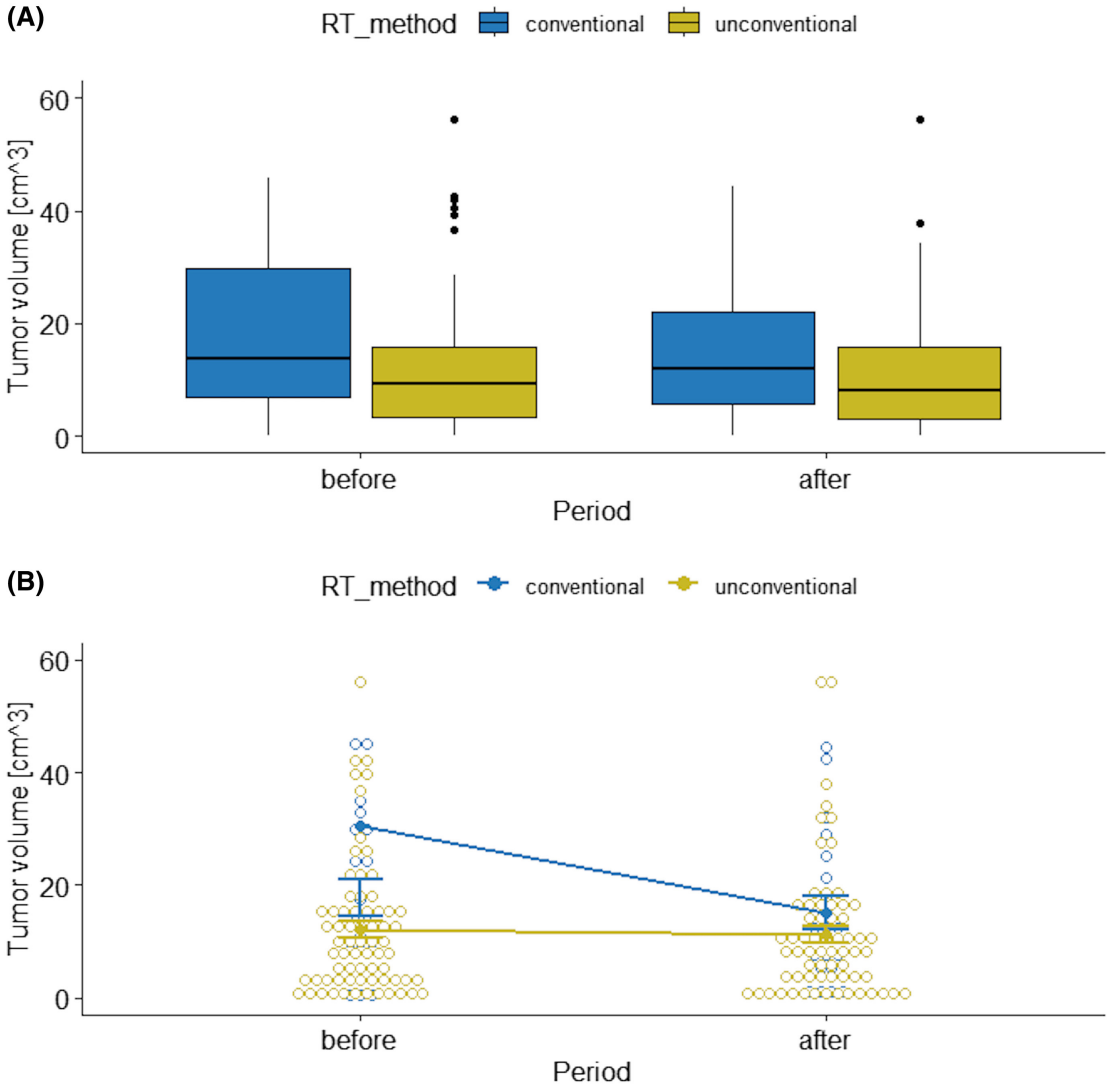
Box plots (A) and interaction plot (B) of tumor volume by RTH method and observation period.

Following the data given in Table 2, there was (30.5−15=) 15.5 cm^3^ reduction of the tumor volume after the treatment of the conventional RTH method, while only less than 1 cm^3^ if using unconventional RTH. To visually inspect the data, the box plots and interaction plots were created (Figure 1A,B, respectively).

Principally, box plots (Figure 1A) show the central tendency, degree of symmetry, range of variation, and potential outliers of a data set. The lower and upper value of the box represents the 25th and 75th percentile for the data, respectively. Thus, 50 percent (median) of the data falls within the box. Additionally, the bottom and top of the whisker is the 25th and 75th percentile minus/plus 1.5 times the interquartile range, correspondingly (any value outside of this range is considered a statistical outlier, and is represented by a dot on the plot).

In turn, an interaction plot (Figure 1B) displays the means at each period of the tumor volume (on the *y*‐axis response variable) for the conventional RTH and has a separate line for the unconventional RTH (on the *x*‐axis of the explanatory variables). Based on these plots in Figure 1, it appears that tumor volumes are different across the two RTH methods for observation periods.

**FIGURE 2:**
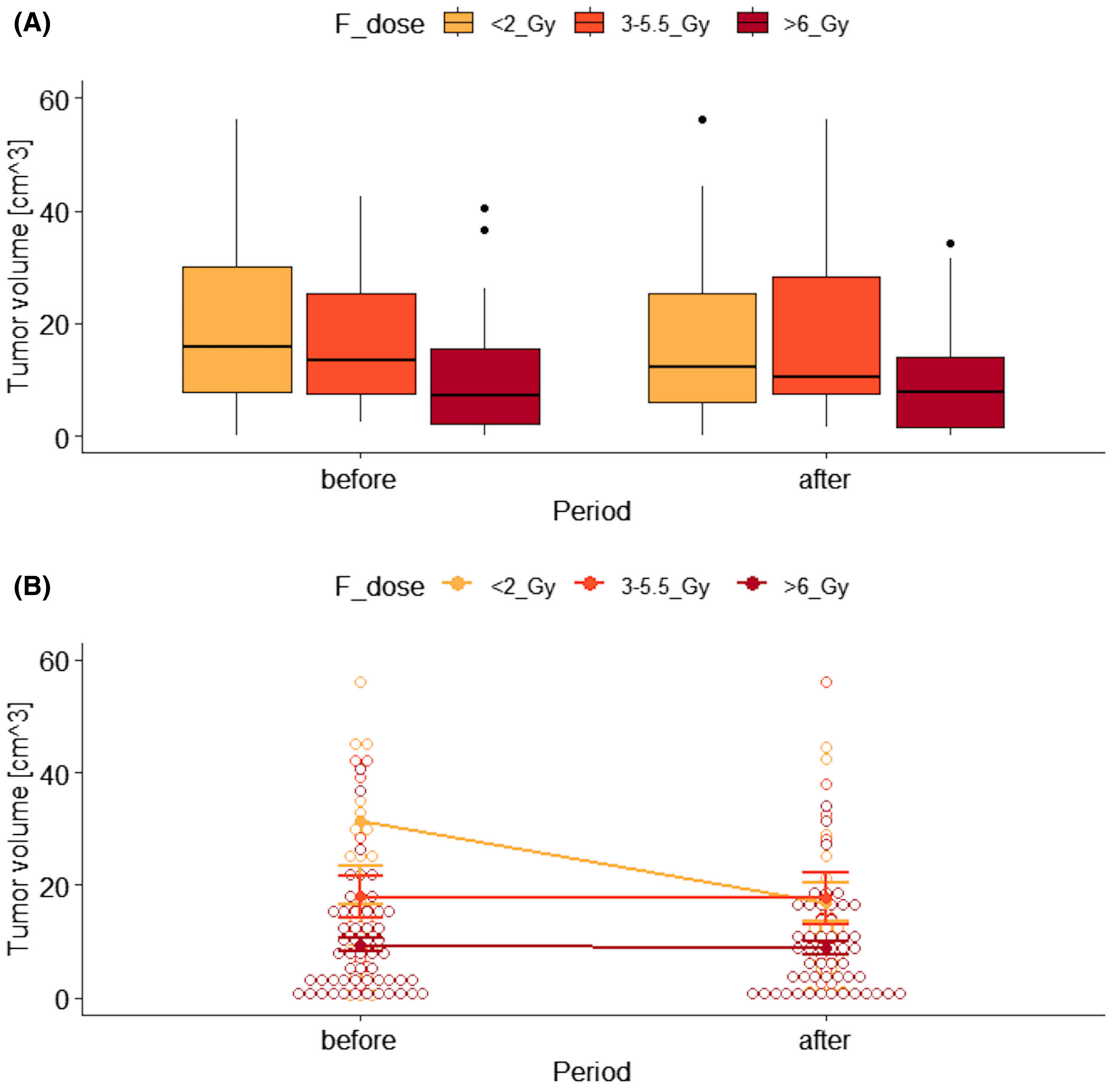
Box plots (A) and interaction plot (B) of tumor volume by RTH fractional dose and observation period.

Based on Table 2, there was (31.4−17=) 14.4 cm^3^ reduction of the tumor volume after the treatment with the lowest RTH fractional dose (<2 Gy), while rather stable using higher doses (>2 Gy). To visually inspect the data, the box plots and interaction plot were created (Figure 2A,B, respectively).

**FIGURE 3:**
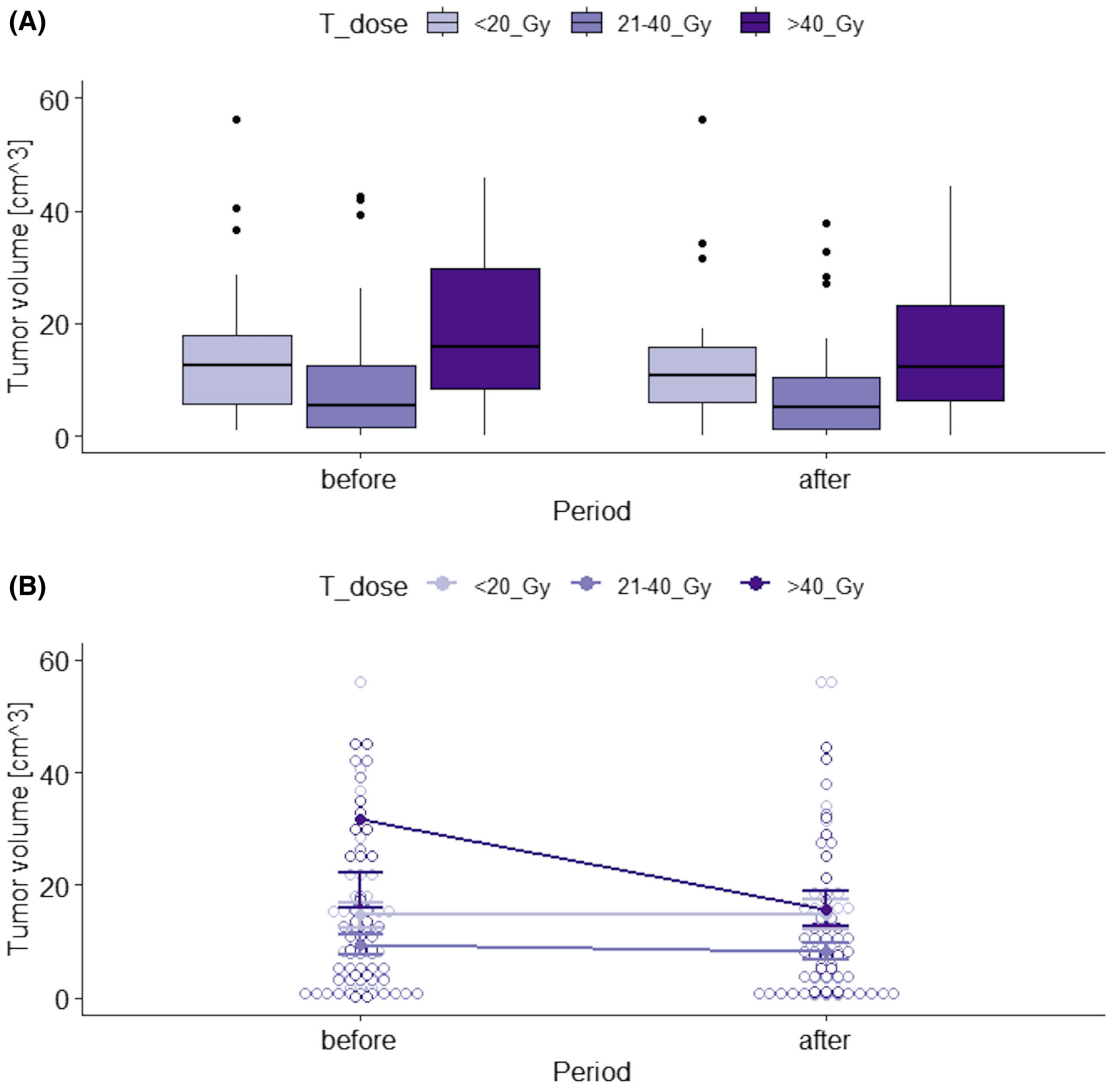
Box plots (A) and interaction plot (B) of tumor volume by RTH total dose and observation period.

Moreover, there was (31.7−15.8=) 15.9 cm^3^ reduction of the tumor volume after the treatment with the highest RTH total dose (>40 Gy), while stable using lower doses (≤40Gy). To visually examine the data, the box plots and interaction plot were created (Figure 3A,B, respectively).

The statistical results of the two‐way ANOVA with interaction (to test three null hypotheses, i.e. that the means of observations grouped, respectively by RTH factor, fractional dose and total dose are the same, and that the means of observations grouped by the period factor are the same, and that there is no interaction between these two factors) is reported in Table 3.

**TABLE 3:** Two‐way ANOVA table with interaction of the RTH methods, fraction dose and total dose and observation periods on tumor volume.

Factor	*p*‐value
RTH method	0.0091
Period	0.0525
RTH method × period	0.0820
Fractional dose	0.0013
Period	0.2051
Fractional dose × period	0.2333
Total dose	0.0055
Period	0.1226
Total dose × period	0.1885

The estimated *p*‐values reported in Table 3 imply to reject a null hypothesis about the lack of influence of the RTH method on the tumor volume (*p* < 0.05), whereas the effect of the observation period is on the border of the statistical significance (*p* < 0.1). Additionally, tumor volume changed differently following the RTH method in time of observation, however, also on the border of the statistical significance (*p* < 0.1). The estimated *p*‐values also indicate an effect of RTH fractional doses (all together) and RTH total doses (all together) on the tumor volume (*p* < 0.05). The impacts of the observation period itself and its interaction with fractional doses and with total doses over time were statistically non‐significant (*p* > 0.05).

Finally, the estimated pairwise contrasts between means of tumor volume (with standard errors = SEs) for RTH methods, fractional dose, total dose and observation period of the groups are given in Table 4. The results given in this table indicate a statistically significant (*p* < 0.05) treatment advantage in the case of using the conventional RTH method, the lowest fractional doses and highest total doses whereas its clinical deficit in patients undergoing unconventional RTH, treated by higher fractional doses (>2 Gy) and lower total doses (<40Gy).

**TABLE 4:** Pairwise contrasts between the means of tumor volume (cm^3^) for RTH methods, fractional dose, total dose, and observation periods.

	Contrast	Mean	SE	*p*‐value
RTH conventional	Before‐after	15.48	7.32	0.0359
RTH unconventional	Before‐after	0.85	4.05	0.8344
Fractional dose <2 Gy	Before‐after	14.44	6.97	0.0399
Fractional dose 3–5.5 Gy	Before‐after	0.348	8.9	0.9689
Fractional dose >6 Gy	Before‐after	0.708	4.53	0.8761
Total dose <20Gy	Before‐after	−0.13	6.01	0.9827
Total dose 21–40Gy	Before‐after	1.16	5.37	0.8301
Total dose >40Gy	Before‐after	15.92	7.37	0.0321

Additionally, in subjective judgment, stabilization, and decreased symptoms after radiotherapy indicated 38 and 22 patients, respectively. Three patients declared deterioration of symptoms. In 13 cases data was not collected. There was no relationship between the method of treatment and the assessment of the effect of it by patients.

## 4 DISCUSSION

Paragangliomas of the H&N are sporadic, in the vast of majority benign tumors. Incidence rates of PGs stay between 0.3 and 1 per 100,000.^38^ Based on Polish National Cancer Registry, only 397 cases of tumors developing from paraganglial tissue, without reporting precise localizations, were diagnosed in 2000–2015.^39^ The presented work collecting 76 patients shows one of the largest groups of patients diagnosed with paraganglioma of the H&N region treated in one center. Treatment of PGs consists of a wide spectrum of possibilities. Surgery, radiotherapy (stereotactic or conventional), embolization, or observation could be used depending on the patient's preferences and the experience of the medical team. Multi‐disciplinary approach, based on consultation with an otolaryngologist, radiation oncologist, endocrinologist, radiologist, and clinical geneticist, is recommended before qualification for any method of treatment.^26^ For asymptomatic patients with an incidentally found paraganglioma, observation with a watchful waiting strategy seems the appropriate solution.^40^ In a retrospective Dutch study, evaluating 157 H&N PGs, motivation for secondary intervention after the wait‐and‐scan period were the following factors: the growth of the tumor (44%), tumor‐induced complications (50%), or patient preference (6%). Ninety percent of analyzed tumors demonstrated growth within 52 months.^41^ This approach allows for to delay of the employment of surgery or radiotherapy and potential related complications. In our group of patients, nine had no symptoms of paraganglioma, however radiotherapy was applied due to the patients' will.

Surgery is the only method of gaining a complete response but multiple feeding arteries and adhesion to large vessels might be causes of difficulties during resection.^42^ Skull base paragangliomas comprise the contraindication for surgical approach due to the risk of incomplete resection. Operation is preferable for smaller tumors when it partially surrounds the artery.^38^ Some researchers suggest that combined surgical treatment with preoperative embolization should be carried out to reduce intraoperative blood loss and operative time during resection.^43^, ^44^ Surgical excision was the main and effective method of treatment for patients with paragangliomas of the H&N in a retrospective cohort study from Michigan.^45^ Unfortunately, about 40% rate of iatrogenic nerve dysfunction was observed after surgery of carotid body paragangliomas. Conversely, conventional and stereotactic radiotherapy used in jugular and vagal paragangliomas had not caused any complications providing good tumor control. The above cited studies show that surgery seems to be a more complicated approach, bounded the higher risk of complications, than radiotherapy. All presented in our work patients were treated with radiotherapy. Twenty‐seven paragangliomas (31% of all 85 paragangliomas) were irradiated after operation. Counterwise, surgery was not needed after radiotherapy in any cases. These data suggest unequivocally that radiotherapy should be considered as a primary method of treatment. Based on the tumor volume and the own experience of the institutions, a patient with paraganglioma may be qualified for conventional or unconventional radiotherapy. In the analysis from 13 institutions of the Rare Cancer Network, researchers presented 81 patients with 82 jugulotympanic and carotid body paragangliomas. Conventional radiotherapy with a median dose of 53 Gy (range 28–70 Gy) was applied in 62 lesions with a median tumor size 30 mm (range 10–150 mm), stereotactic radiotherapy in 2–5 fractions to a total dose of 24 Gy (range 19–30 Gy) was used in 13 lesions and radiosurgery realized in one fraction with a median dose 12 Gy (range 12–15 Gy) was implemented in 7 lesions. The median tumor size in the group of patients treated with unconventional schemes was 48 mm (range 17–92 mm). During median follow‐up of 48 months, local control was achieved in 90%. Regression was identified in 22 lesions, progression occurred in 11 patients. Tolerance of the treatment was acceptable, only 3 and 5 patients reported severe acute and late toxicity, respectively. No disease progression and late toxicity were found after stereotactic radiotherapy.^46^ In the Italian publication, authors described patients with H&N paraganglioma after stereotactic radiotherapy. The single‐fraction radiosurgery (range 11–13 Gy) was given in 7 lesions (mean volume 4.0 cc) and multisession radiotherapy to total dose in the range 20–30 Gy delivered in 3–5 fractions was applied in 14 lesions (mean volume 18.9 cc). In six cases after multisession radiotherapy tumor shrinkage was observed as in only one case after single‐fraction radiosurgery. Tolerance of the treatment was good in all cases. No progression was stated during the mean 46.3 months of follow‐up. Neurologic improvement or stagnations of symptoms were reported in 45% and 40% of patients, respectively.^47^ Excellent local control without severe complication in the group of 149 patients during a median follow‐up of 10.6 years was reported in Mendenhall's paper. Radiotherapy was conducted to a total dose of 35–61.5 Gy. The most common scheme was conventional radiotherapy to 45 Gy in 25 fractions. Progression developed only in 6 patients after radiotherapy.^12^ The interesting volumetric analysis was conducted in a German paper in 40 patients with H&N paraganglioma after fractionated stereotactic radiotherapy to a median total dose of 54 Gy in single doses of 1.8 or 2 Gy. After 24.6 months of follow‐up mean tumor volume shrank down to 86.1% in correlation to initial volume. Transient enlargement in the range of 129.6%–151.2% was noted in three cases. 7.5% of patients informed about the worsening of symptoms and the remaining experienced improvement or stabilization.^48^ In the Turkish publication, 54% of paragangliomas based on RECIST criteria had partial regression after robotic radiosurgery treated to a total dose of 21–30 Gy in 3–5 fractions. Other 46% of tumors were considered as stable disease. Initial tumor volume was in the range 5.3–113.8 cc. No acute or late toxicity was documented.^49^ Regarding to above‐mentioned publications, our study also proved the high efficacy and safety of all used schemes of radiotherapy. Regression of the tumor volume was the most noticeable with the application fractional dose ≤2 Gy and total dose >40 Gy in the largest paragangliomas, however without advantage compared to other fractionation methods on the improvement of reported symptoms.

## 5 CONCLUSIONS

Radiotherapy is a useful and well‐tolerated method of treatment notably after administration conventional scheme to a total dose >40 Gy, where the most significant depletion of the volume of paragangliomas was noted. The precise establishment of the most effective radiotherapy scheme should be determined in randomized clinical trials.

## AUTHOR CONTRIBUTIONS

**Paweł J. Polanowski:** Conceptualization (lead); data curation (equal); formal analysis (lead); investigation (lead); methodology (lead); writing – original draft (lead); writing – review and editing (lead). **Agnieszka R. Kotecka‐Blicharz:** Conceptualization (equal); formal analysis (equal); writing – original draft (equal); writing – review and editing (equal). **Andrzej Tukiendorf:** Conceptualization (equal); data curation (equal); formal analysis (equal). **Natalia J. Amrogowicz:** Data curation (equal). **Aleksandra M. Nasiek:** Data curation (equal); formal analysis (equal); writing – review and editing (equal). **Agnieszka Pietruszka:** Formal analysis (equal); methodology (equal); writing – review and editing (equal). **Katarzyna M. Polanowska:** Formal analysis (equal); methodology (equal); writing – review and editing (equal). **Krzysztof A. Składowski:** Supervision (equal).

## FUNDING INFORMATION

This research have not received any external funding.

## CONFLICT OF INTEREST STATEMENT

All authors declare no conflict of interest.

## ETHICS STATEMENT

All patients included in the analysis were qualified for treatment during the medical case conference. Schemes of fractionation were chosen based on the tumor size, protocols and literature. This study was not presented for the Ethics Committee's opinion due to the gained information about the lack of indispensability (it is not a clinical trial or experiment).

## CONSENT

All patients signed up for the consent of treatment before the beginning. All patients had the possibility to ask questions and he gained answers for all issues.

## PRÉCIS

The results show a statistically significant (*p* < 0.05) treatment advantage for conventional RTH, the lowest fractional doses and highest total doses, while its clinical deficit in patients undergoing unconventional RTH, treated with higher fractional doses (>2 Gy) and lower total doses (<40 Gy).

## Data Availability

The datasets used and analyzed during the current study are available from the corresponding author on reasonable request.
